# Association Between Intradialytic Foot Temperature Gradients and Severe Peripheral Artery Disease in Patients Undergoing Hemodialysis: Prospective Cohort Study

**DOI:** 10.2196/96782

**Published:** 2026-11-06

**Authors:** Donna Shu-Han Lin, Chih-Cheng Wu, Chung-Wei Yang, Jen-Kuang Lee, Chien-Yi Hsu

**Affiliations:** 1 Department of Internal Medicine, Division of Cardiology, Shin Kong WHS Memorial Hospital, Taipei, Taiwan; 2 Department of Internal Medicine, Division of Cardiology, National Taiwan University, Hsin-Chu Branch, Hsinchu, Taiwan; 3 Department of Internal Medicine, College of Medicine, National Taiwan University Hospital, Taipei, Taiwan; 4 Institute of Biomedical Engineering, National Tsing-Hua University, Hsinchu, Taiwan; 5 Institute of Cellular and System Medicine, National Health Research Institutes, Zhunan, Taiwan; 6 Department of Internal Medicine, Division of Nephrology, National Taiwan University Hospital, Hsin-Chu Branch, Hsinchu, Taiwan; 7 Department of Internal Medicine, Division of Cardiology, National Taiwan University Hospital, Taipei, Taiwan; 8 Department of Laboratory Medicine, National Taiwan University College of Medicine, Taipei, Taiwan; 9 Cardiovascular Center, National Taiwan University Hospital, Taipei, Taiwan; 10 Telehealth Center, National Taiwan University Hospital, Taipei, Taiwan; 11 Taipei Heart Institute, Taipei Medical University, No. 252, Wuxing St, Taipei, Taiwan, 110, Taiwan, 886 27361661; 12 Division of Cardiology, Department of Internal Medicine, School of Medicine, College of Medicine, Taipei Medical University, Taipei, Taiwan

**Keywords:** mobile health, mHealth, sensors, peripheral artery disease, hemodialysis, remote patient monitoring

## Abstract

**Background:**

Peripheral artery disease (PAD) is prevalent in hemodialysis (HD) patients but remains difficult to diagnose due to unreliable ankle-brachial index (ABI) measurements caused by vascular calcification. We hypothesized that monitoring foot temperatures and their changes during HD, provoked by hemodynamic shifts, could serve as a novel diagnostic tool for detecting severe PAD.

**Objective:**

This study aims to evaluate the diagnostic utility of intradialytic foot temperature monitoring using wearable mobile health (mHealth) sensors for detecting severe PAD in patients undergoing HD.

**Methods:**

In this prospective observational study, we recruited 109 patients undergoing chronic maintenance HD. Skin temperatures were monitored continuously at the instep and sole during HD sessions using wearable sensors (Temp Pal, iWEECARE Co, Ltd), which are wireless, nanochip-based mHealth thermometers with ±0.05 °C accuracy. The diagnostic performance of temperature parameters, specifically the temperature difference between the wrist and foot at the end of HD, was evaluated against vascular duplex ultrasound (DUS). Severe PAD was defined as abnormal (monophasic or absent) flow in the distal posterior tibial artery (PTA) or anterior tibial artery (ATA).

**Results:**

Patients with abnormal tibial flow on DUS exhibited significantly different temperature gradients between the wrist and foot at the end of dialysis compared to those with normal flow. Receiver operating characteristic (ROC) analysis demonstrated that the wrist-foot temperature difference at the sole effectively discriminated abnormal ATA flow (area under the curve [AUC] 64.5%, 95% CI 57.8%‐70.9%) and abnormal PTA flow (AUC 62.0%, 95% CI 55.2%‐68.5%). Notably, an instep wrist-foot temperature difference cutoff of –2.2 °C or less yielded a specificity of 97.6% (95% CI 94.0%-99.3%) for detecting abnormal PTA flow.

**Conclusions:**

Intradialytic foot temperature monitoring, particularly the temperature gradient between the wrist and foot, significantly correlates with severe tibial artery flow abnormalities. This method offers an accessible, noninvasive screening adjunct to the ABI for detecting severe PAD in the HD population. These findings indicate an association rather than a standalone diagnostic test; given the modest discriminative ability, external validation is warranted.

## Introduction

Patients with end-stage renal disease (ESRD) are at elevated risk of multivascular cardiovascular disease, including coronary artery disease (CAD), cerebrovascular disease, and peripheral artery disease (PAD) [1,2], compared to the general population. In addition, the presentation of PAD in patients undergoing dialysis manifests a worse prognosis than that in patients without ESRD, with greater risk of major adverse limb events, including amputation or hospitalization for chronic limb-threatening ischemia (CLTI), and mortality [3-6]. Indeed, patients with ESRD receiving dialysis are at even higher risk of PAD and adverse limb outcomes than those in earlier stages of chronic kidney disease. As with most diseases, earlier detection is pivotal to the optimal management of these patients. However, diagnosis of PAD remains challenging in the hemodialysis (HD) population for several reasons. First, symptoms are often mild, atypical, or even absent in this group due to limited ambulation or concomitant neuropathy [7]. Second, the severe vascular calcification that is commonly seen in patients with ESRD often leads to falsely elevated ankle-brachial index (ABI) measurements due to noncompressible vessels [8]. Third, the presence of below-the-ankle disease in renal-diabetic foot ulcers is associated with higher risk of treatment failure [9] but is less likely to be detected using conventional ABI or vascular duplex measurements in the tibial arteries. Several noninvasive modalities are used to assess PAD, including the ABI, toe-brachial index, Doppler waveform analysis, duplex ultrasonography, and computed tomographic or magnetic resonance angiography [10]. Infrared thermography, which maps skin-temperature variation as a surrogate for perfusion, has also been applied to identify ischemic changes, although it requires dedicated cameras and is sensitive to ambient conditions [11,12].

Foot temperature monitoring has previously been advocated as a means of surveillance for the development of diabetic foot ulcers [13-15]. This was based on the rationale that inflammation usually precedes overt ulceration in neuropathic ulcers, which can be detected by an increase in local foot temperature. However, for ischemic ulcers seen in PAD, temperatures are usually low. We previously demonstrated that an increase in foot temperature of 2 °C or greater after endovascular therapy (EVT) for chronic foot ulcers in the absence of active infection was associated with better wound healing compared to those with a foot temperature increase of less than 2 °C after EVT [16]. As yet, how temperatures relate to outcomes in asymptomatic patients is uncertain. We hypothesized that foot temperatures in asymptomatic patients also relate to the degree of ischemia and may provide significant diagnostic value. In addition, in patients with ESRD whose ABIs are often falsely negative due to noncompressible vessels, foot temperatures may have higher sensitivity in diagnosing PAD than conventional ABIs and offer greater accessibility than vascular duplex ultrasound (DUS).

Because symptoms related to PAD often manifest in patients with ESRD during their HD sessions due to the significant fluid shifts and hemodynamic changes, we initiated a prospective study that investigated the associations among ABI, DUS measurements, and intradialytic foot temperatures. Our goal was to explore the diagnostic value of intradialytic foot temperatures in identifying severe PAD, potentially offering a readily accessible means of early detection in this vulnerable patient group.

## Methods

### Study Cohort

This prospective, observational cohort study was conducted at 2 tertiary centers in Taiwan: National Taiwan University Hospital, Hsinchu Branch, and Shin Kong Wu Ho-Su Memorial Hospital. Patients with ESRD undergoing maintenance HD were screened for inclusion. Exclusion criteria included age under 20 years, prior lower-limb amputations (minor or major), and refusal to provide informed consent. Each lower limb was evaluated as an independent unit. Following enrollment, data on participant characteristics, biochemical profiles, and ABI measurements were collected. The presence of severe PAD was confirmed using DUS as the gold standard, defined by monophasic or absent flow in the distal posterior tibial artery (PTA) and distal anterior tibial artery (ATA) at 5 cm above the ankle.

### Ethical Considerations

This study was approved by the Institutional Review Board (IRB) of both the National Taiwan University Hospital, Hsinchu Branch (IRB number 111‐105-E), and Shin Kong Wu Ho-Su Memorial Hospital (IRB number 20230702D); written informed consent was obtained from all participants.

### Data Collection

Following study enrollment, a systematic review of electronic medical records and baseline clinical assessments of the participants was conducted. Demographic information, including age and sex, and a detailed history of comorbid conditions such as hypertension, diabetes mellitus (DM), hyperlipidemia, CAD, congestive heart failure, and prior cerebrovascular or myocardial events were recorded. Current medication profiles were also documented, specifically the use of erythropoietin, vitamin D analogs, antihypertensive agents, and iron supplements. Comprehensive laboratory data were gathered, including complete blood counts, serum albumin, and markers of dialysis adequacy such as predialysis and postdialysis blood urea nitrogen and Kt/V. Additionally, we recorded metabolic indicators including fasting blood glucose, lipid panels, and bone mineral markers such as calcium, phosphorus, and intact parathyroid hormone levels. As part of the initial vascular screening, the ABI was measured for each limb.

### Foot Temperature Measurements

To record skin temperatures, contact thermometers were placed on the instep and sole of both feet, as well as the wrist of the nonaccess arm (Figures 1 and 2). The instep (dorsal midfoot) overlies the anterior tibial and dorsalis pedis angiosomes, whereas the sole reflects the posterior tibial angiosome; monitoring both sites therefore samples the 2 principal tibial-artery territories assessed by DUS, and these acral sites are among the earliest to display perfusion-related thermal changes. We used Temp Pal (iWEECARE Co Ltd), a wireless, mobile health (mHealth)–based thermometer system that allows for simultaneous, multisite continuous monitoring. Five devices were paired via Bluetooth to a single iOS mobile device running the Temp Pal app, which captured and uploaded continuous temperature data to a cloud-based server for export. This system uses an autocalibration algorithm to ensure a high degree of measurement reliability, maintaining accuracy and precision within ±0.05 °C. Baseline body temperature was measured at the start and end of each HD session using an infrared ear thermometer, with simultaneous readings obtained from the skin sensors. Throughout the entire procedure, skin temperatures at the wrist and both feet were monitored continuously to capture fluctuations related to intradialytic hemodynamic shifts. Furthermore, clinical parameters, including dialysate temperature, blood pressure, and heart rate, were systematically recorded during the session.

**Figure 1.**
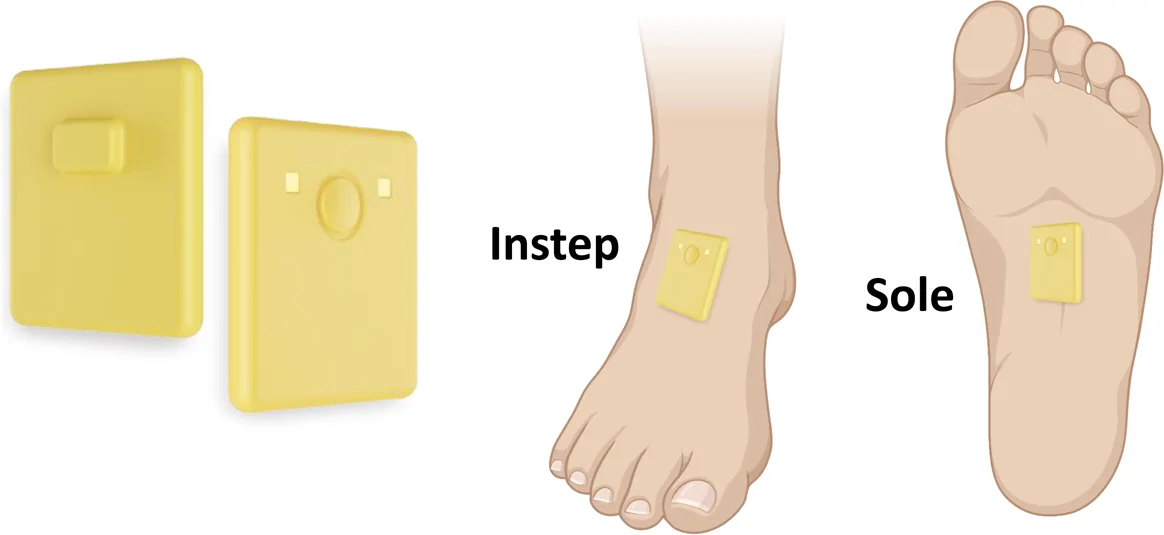
Schematic of foot temperature monitoring using Temp Pal nanochip-based thermometer.

**Figure 2.**
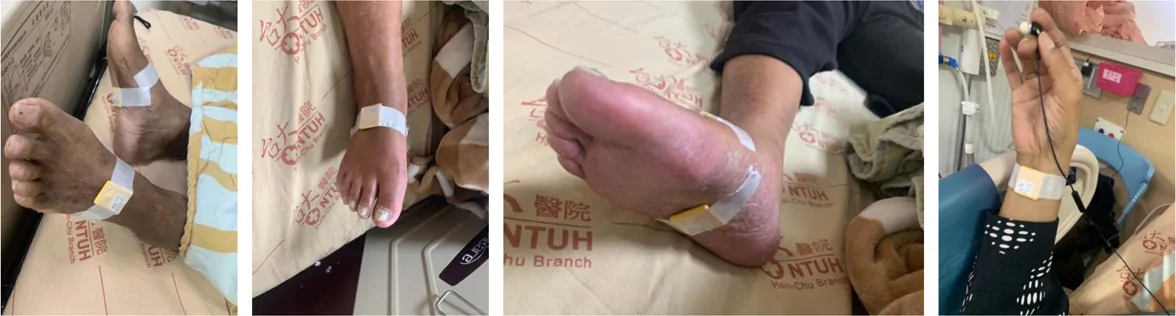
Example photos showing intradialytic foot and wrist temperature monitoring with Temp Pal nanochip-based thermometer.

### Vascular DUS Measurements

To evaluate the presence and severity of PAD, all participants underwent DUS performed by experienced technicians. DUS served as the gold standard for this study, specifically focusing on flow patterns in the tibial arteries. Measurements were obtained from the distal PTA and ATA at a standardized location 5 cm above the ankle. Severe PAD was defined by the identification of monophasic or absent flow in either the distal PTA or ATA. These flow patterns were contrasted with normal (triphasic or biphasic) flow to categorize the limbs for comparative analysis of intradialytic temperature shifts.

### Statistical Analysis

Inferential analyses were conducted at the limb level; 109 patients contributed 218 limbs eligible for analysis. Temperature parameters were compared between limbs with normal vs abnormal distal PTA/ATA flow using generalized estimating equation (GEE) with a normal distribution and an identity link, accounting for the intrapatient correlation between the 2 limbs of the same individual by using robust (empirical) standard errors. We further performed multivariable GEE analyses to investigate the independent associations of each temperature parameter and ABI value with abnormal distal PTA/ATA flow. The models were adjusted for age, DM, CAD, congestive heart failure, Kt/V (Daugirdas formula), and calcium × phosphorus product. We intentionally limited the adjustment to 6 confounding variables to avoid model overfitting, given that the number of events was 51 for abnormal distal PTA flow and 48 for abnormal distal ATA flow.

Discriminative performance of abnormal distal PTA/ATA flow was evaluated using receiver operating characteristic (ROC) curve analysis, with area under the curve (AUC) calculated as the primary metric. The 95% CI for the AUC was derived using DeLong nonparametric method. When the AUC was statistically significant (ie, the 95% CI did not include 0.5), the optimal cutoff was determined using the Youden index to balance sensitivity and specificity. To determine if temperature monitoring offers incremental diagnostic value beyond the ABI, the statistical significance of differences between the respective AUC values was assessed via DeLong test. Statistical significance was defined as a 2-sided *P* value of less than .05. All analyses were conducted with MedCalc Statistical Software (version 20.019; MedCalc Software).

## Results

### Baseline Characteristics of the Study Cohort

The study included 109 patients with ESRD undergoing maintenance HD. The mean age of the participants was 65.2 (SD 11.9) years, and 56.9% (n=62) were male participants. Comorbid conditions were highly prevalent in this cohort, with hypertension (n=89, 81.7%) and DM (n=61, 55.9%) being the most frequent, followed by gout (n=29, 26.6%), CAD (n=26, 23.9%), and congestive heart failure (n=25, 22.9%). A smaller proportion of patients had a history of malignancy (n=6, 5.5%), cerebrovascular accidents (n=4, 3.7%), and myocardial infarctions (n=2, 1.8%). Regarding pharmacological management, the majority of patients were receiving erythropoietin (n=97, 88.9%) and iron supplements (n=84, 77.1%), while 58.7% (n=64) were on antihypertensive medications. Laboratory assessments revealed a mean hemoglobin of 11.6 (SD 1.2) g/dL and mean albumin of 3.99 (SD 0.37) g/dL. Dialysis adequacy was reflected by a mean Daugirdas Kt/V of 1.62 (SD 0.21). Biochemical markers related to bone and mineral metabolism showed a mean calcium of 8.92 (SD 0.98) mg/dL, mean phosphorus of 5.03 (SD 1.35) mg/dL, and median intact parathyroid hormone level of 290.7 (IQR 106.1-570.8) pg/mL. Iron status was characterized by a mean transferrin saturation of 27.9% (SD 11.8%) and median ferritin level of 567 (IQR 333-738) ng/mL. Detailed demographic, clinical, and biochemical data are summarized in Table 1.

**Table 1.** Demographics and characteristics of the 109 patients.

Variable	Descriptive statistics
Demographics	
Age (y), mean (SD)	65.2 (11.9)
Sex (male), n (%)	62 (56.9)
Comorbid condition, n (%)	
Hypertension	89 (81.7)
Diabetes mellitus	61 (55.9)
Hyperlipidemia	47 (43.1)
Coronary artery disease	26 (23.9)
Congestive heart failure	25 (22.9)
Chronic obstructive pulmonary disease	1 (0.9)
Gout	29 (26.6)
Chronic hepatitis	11 (10.1)
Neuropathy	2 (1.8)
Malignancy	6 (5.5)
History of prior event, n (%)	
Myocardial infarction	2 (1.8)
Cerebrovascular accident	4 (3.7)
Gastrointestinal bleeding	0 (0)
Parathyroidectomy	2 (1.8)
Medication, n (%)	
Erythropoietin	97 (88.9)
Vitamin D	11 (10.1)
Antihypertensive drugs	64 (58.7)
Iron supplements	84 (77.1)
Complete blood count, mean (SD)	
White blood cell count (1000/μL)	6.5 (2.1)
Red blood cell (1000/μL)	3.6 (0.5)
Hemoglobin (g/dL)	11.6 (1.2)
Hematocrit (%)	35.1 (3.5)
Mean corpuscular volume (fL)	93.5 (7.4)
Platelet (1000/μL)	168.1 (58.0)
Biochemistry	
Albumin (g/dL), mean (SD)	3.99 (0.37)
Alanine aminotransferase (U/L), mean (SD)	18.5 (10.8)
Aspartate aminotransferase (U/L), mean (SD)	16.6 (13.1)
Alkaline phosphatase (U/L), median (IQR)	70 (57-84)
Total cholesterol (mg/dL), mean (SD)	147.9 (36.0)
Triglyceride (mg/dL), median (IQR)	138.5 (90.5-207.0)
Fasting blood glucose (mg/dL), mean (SD)	136.6 (48.4)
Predialysis blood urea nitrogen (mg/dL), mean (SD)	83.2 (24.6)
Postdialysis blood urea nitrogen (mg/dL), mean (SD)	21.6 (7.8)
Creatinine (mg/dL), mean (SD)	11.7 (2.7)
Uric acid (mg/dL), mean (SD)	7.6 (1.9)
Sodium (mEq/L), mean (SD)	137.1 (3.3)
Potassium (mEq/L), mean (SD)	4.7 (0.70)
Calcium (mg/dL), mean (SD)	8.92 (0.98)
Phosphorus (mg/dL), mean (SD)	5.03 (1.35)
Urea reduction ratio, Kt/V, mean (SD)	58.0 (30.8)
Gotch, Kt/V, mean (SD)	1.37 (0.18)
Daugirdas, Kt/V, mean (SD)	1.62 (0.21)
Iron (ug/dL), mean (SD)	64.3 (27.3)
Unsaturated iron-binding capacity (ug/dL), mean (SD)	173.3 (47.2)
Total iron binding capacity (ug/dL), mean (SD)	231.7 (42.2)
Ferritin (ng/mL), median (IQR)	567 (333-738)
Transferrin saturation (%), mean (SD)	27.9 (11.8)
Intact parathyroid hormone (pg/mL), median (IQR)	290.7 (106.1-570.8)
Calcium × phosphorus, mean (SD)	45.6 (13.1)

### Foot Temperature Parameters and ABI in Limbs With and Without Severe PAD

A total of 218 limbs were evaluated to analyze the relationship between distal arterial flow and various physiological parameters (Table 2). The parameters analyzed comprised the ABI and the temperature parameters, namely, wrist-to-foot and ear-to-foot temperature differences at the end of HD and absolute foot temperature before and at the end of HD. Limbs with abnormal flow in either the distal PTA or ATA exhibited significantly lower ABI values compared to those with normal flow (mean 0.90, SD 0.26 vs mean 1.13, SD 0.20 for PTA; mean 0.89, SD 0.27 vs mean 1.13, SD 0.200 for ATA; both *P*<.001). However, it is noteworthy that despite these significant differences, limbs with confirmed severe PAD still exhibited mean ABI values as high as 0.89 (SD 0.27) to 0.90 (SD 0.26), which border the clinically normal range and highlight the limitations of ABI in representing disease severity in this population.

**Table 2.** Comparison of temperature parameters in limbs with normal and abnormal distal posterior tibial artery (PTA)/anterior tibial artery (ATA) flow.

Variable	Limbs (N=218), mean (SD)	Distal PTA flow			Distal ATA flow		
		Abnormal (n=51), mean (SD)	Normal (n=167), mean (SD)	*P* value	Abnormal (n=48), mean (SD)	Normal (n=170), mean (SD)	*P* value
Ankle-brachial index (n=203)	1.08 (0.23)	0.90 (0.26)	1.13 (0.20)	<.001	0.89 (0.27)	1.13 (0.20)	<.001
Instep (°C)							
Temperature difference between the wrist and foot at the end of the HD^a^ session	1.50 (2.68)	0.75 (3.15)	1.73 (2.48)	.051	0.61 (2.91)	1.75 (2.57)	.046
Temperature difference between the ear and foot at the end of the HD session	5.38 (2.54)	4.92 (2.66)	5.52 (2.50)	.10	4.69 (2.59)	5.57 (2.50)	.09
Foot temperature before the HD session	30.51 (2.80)	30.73 (2.77)	30.45 (2.82)	.45	30.62 (2.59)	30.48 (2.87)	.99
Foot temperature at the end of the HD session	30.69 (2.50)	31.14 (2.66)	30.55 (2.44)	.11	31.41 (2.55)	30.48 (2.46)	.08
Sole (°C)							
Temperature difference between the wrist and foot at the end of the HD session	1.95 (2.73)	1.05 (3.05)	2.23 (2.57)	.02	0.81 (2.51)	2.27 (2.71)	.006
Temperature difference between the ear and foot at the end of the HD session	5.83 (2.68)	5.22 (2.64)	6.02 (2.68)	.04	4.90 (2.42)	6.10 (2.70)	.03
Foot temperature before the HD session	30.17 (2.67)	30.29 (2.69)	30.13 (2.67)	.48	30.44 (2.47)	30.09 (2.72)	.33
Foot temperature at the end of the HD session	30.23 (2.60)	30.84 (2.63)	30.05 (2.58)	.04	31.20 (2.36)	29.96 (2.61)	.02

a HD: hemodialysis.

Regarding intradialytic temperature dynamics, the temperature difference between the wrist and the foot at the end of the HD session was significantly smaller in limbs with abnormal flow (Table 2). Specifically, at the sole, the wrist-foot temperature difference was mean 1.05 (SD 3.05) °C in the abnormal PTA group compared to mean 2.23 (SD 2.57) °C in the normal group (*P*=.02). A similar pattern was observed at the instep, reaching statistical significance for ATA flow (*P*=.046) but showing only a borderline association for PTA flow (*P*=.051). While foot temperatures before the HD session did not differ significantly between groups, the foot temperature at the end of the session tended to be higher in limbs with abnormal ATA flow compared to those with normal flow (mean 31.41, SD 2.55 °C vs mean 30.48, SD 2.46 °C at the instep; *P*=.08; mean 31.20, SD 2.36 °C vs mean 29.96, SD 2.61 °C at the sole; *P*=.02).

Additionally, a multivariable GEE model, adjusting for age, DM, CAD, congestive heart failure, Kt/V (Daugirdas formula), and the calcium × phosphorus product, revealed that an increased ABI was significantly associated with a reduced risk of abnormal distal PTA flow (odds ratio [OR] 0.73, 95% CI 0.63‐0.83) and ATA flow (OR 0.71, 95% CI 0.59‐0.85; Table 3). Moreover, wrist-to-foot temperature differences at the end of the HD session, evaluated at the instep and sole, respectively, were significantly associated with abnormal distal PTA flow at the sole (OR 0.81, 95% CI 0.68‐0.97), with a borderline, nonsignificant association at the instep (OR 0.84, 95% CI 0.70‐1.003), and with abnormal distal ATA flow at both the instep (OR 0.84, 95% CI 0.71‐0.99) and sole (OR 0.81, 95% CI 0.70‐0.95). A similar pattern of results was observed for foot temperature at the end of the HD session.

**Table 3.** Associations of each temperature parameter and the ankle-brachial index (ABI) with abnormal distal posterior tibial artery (PTA)/anterior tibial artery (ATA) flow using a generalized estimating equation model^a^.

Variable	Distal PTA flow		Distal ATA flow	
	OR^b^ (95% CI)	*P* value	OR (95% CI)	*P* value
ABI (n=203)	0.73 (0.63‐0.83)	<.001	0.71 (0.59‐0.85)	<.001
Instep				
Temperature difference between the wrist and foot at the end of the HD^c^ session	0.84 (0.70‐1.003)	.053	0.84 (0.71‐0.99)	.04
Temperature difference between the ear and foot at the end of the HD session	0.86 (0.75‐0.995)	.04	0.83 (0.71‐0.98)	.03
Foot temperature before the HD session	1.10 (0.93‐1.31)	.27	1.06 (0.89‐1.26)	.53
Foot temperature at the end of the HD session	1.16 (0.99‐1.35)	.07	1.21 (1.02‐1.44)	.03
Sole				
Temperature difference between the wrist and foot at the end of the HD session	0.81 (0.68‐0.97)	.02	0.81 (0.70‐0.95)	.008
Temperature difference between the ear and foot at the end of the HD session	0.84 (0.73‐0.97)	.02	0.82 (0.70‐0.95)	.008
Foot temperature before the HD session	1.11 (0.93‐1.32)	.27	1.14 (0.93‐1.40)	.20
Foot temperature at the end of the HD session	1.20 (1.02‐1.40)	.03	1.24 (1.06‐1.46)	.008

a Adjusted for age, diabetes mellitus, coronary artery disease, congestive heart failure, Daugirdas Kt/V, and calcium × phosphorus.

b OR: odds ratio.

c HD: hemodialysis.

### Diagnostic Performance of Foot Temperature Parameters in Discriminating an ABI Value of Less Than 0.9

The diagnostic utility of intradialytic temperature monitoring was evaluated for its ability to predict an ABI value of less than 0.9 (Table 4). ROC analysis revealed that the temperature difference between the wrist and foot at the end of the HD session was the most effective parameter for this purpose. At the instep, this gradient achieved an AUC of 64.5% (95% CI 57.5%‐71.0%). Similarly, the gradient measured at the sole demonstrated a significant AUC of 64.2% (95% CI 57.2%‐70.8%; Figure 3). For the instep measurement, an optimal cutoff of 1.25 °C or less yielded a sensitivity of 63.2% (95% CI 46.0%‐78.2%) and a specificity of 60.0% (95% CI 52.1%‐67.5%). At the sole, a cutoff of 2.83 °C or less provided a higher sensitivity of 81.6% (95% CI 65.7%‐92.3%) but lower specificity of 42.4% (95% CI 34.8%‐50.3%). Other parameters, including absolute foot temperatures before or after HD and the ear-foot temperature gradient, did not reach statistical significance in discriminating limbs with an ABI value of less than 0.9 (Table 4).

**Figure 3.**
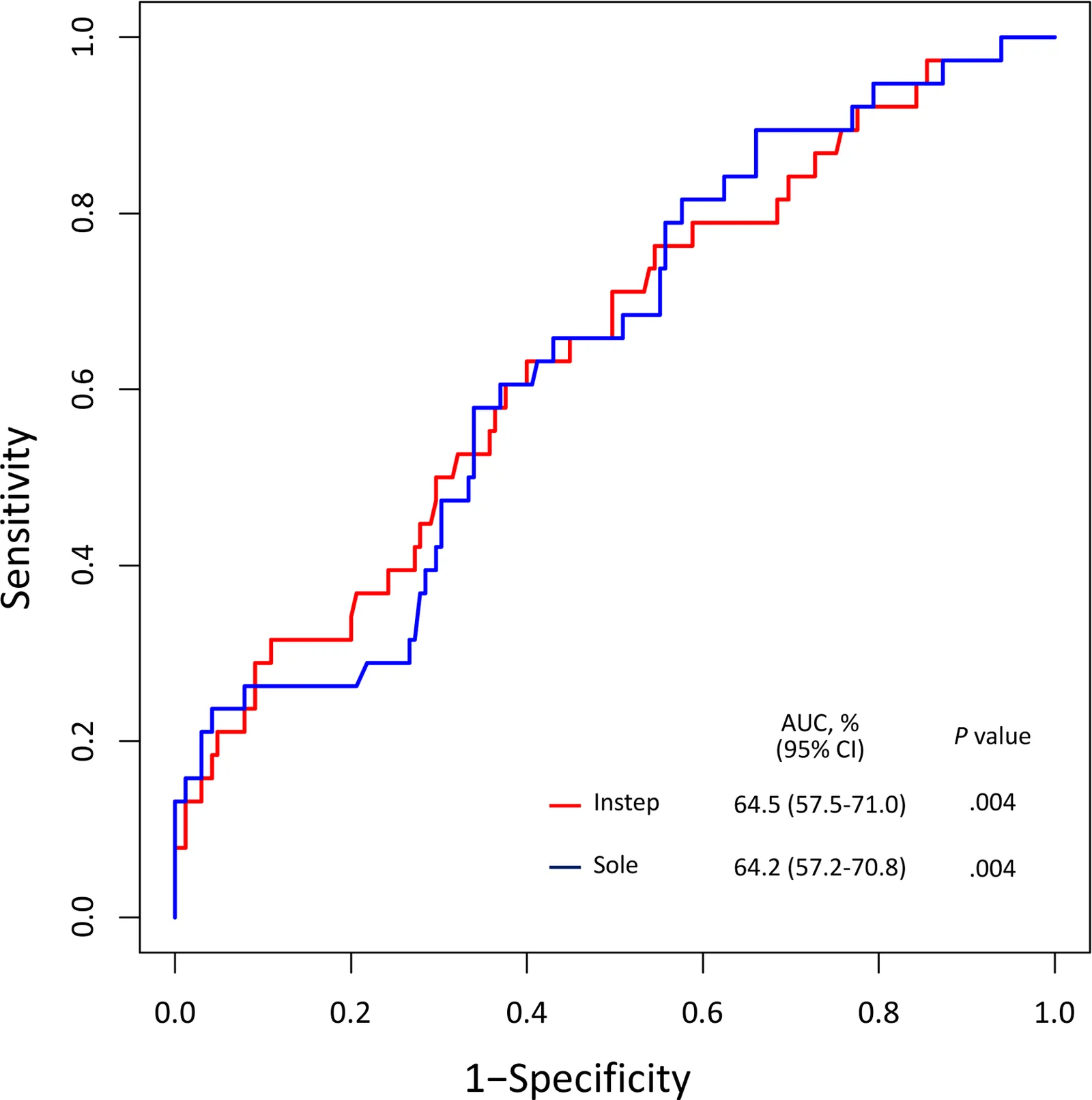
Receiver operating characteristic curves for wrist-to-foot temperature differences measured at the end of the hemodialysis session, at the instep and sole, for the discrimination of ankle-brachial index value of less than 0.9. AUC: area under the curve.

**Table 4.** Summary of temperature parameters of interest for discriminating limbs with an ankle-brachial index value of less than 0.9.

Position/parameter	AUC^a^, % (95% CI)	Cutoff, °C^b^	Sensitivity, % (95% CI)	Specificity, % (95% CI)
Instep				
Temperature difference between the wrist and foot at the end of the HD^c^ session	64.5 (57.5‐71.0)^d^	≤1.25	63.2 (46.0‐78.2)	60.0 (52.1‐67.5)
Temperature difference between the ear and foot at the end of the HD session	53.2 (46.1‐60.3)	≤3.71	39.5 (24.0‐56.6)	72.7 (65.3‐79.4)
Foot temperature before the HD session	50.9 (43.8‐58.0)	≤29.4	18.4 (7.7‐34.3)	67.3 (59.5‐74.4)
Foot temperature at the end of the HD session	54.2 (47.1‐61.2)	>31.12	55.3 (38.3‐71.4)	60.0 (52.1‐67.5)
Sole				
Temperature difference between the wrist and foot at the end of the HD session	64.2 (57.2‐70.8)^d^	≤2.83	81.6 (65.7‐92.3)	42.4 (34.8‐50.3)
Temperature difference between the ear and foot at the end of the HD session	53.9 (46.8‐60.9)	≤4.25	44.7 (28.6‐61.7)	71.5 (64.0‐78.3)
Foot temperature before the HD session	50.5 (43.4‐57.6)	>32.15	13.2 (4.4‐8.1)	70.9 (63.3‐77.7)
Foot temperature at the end of the HD session	55.2 (48.1‐62.2)	>31.58	47.4 (31.0‐64.2)	70.9 (63.3‐77.7)

a AUC: area under the curve.

b According to the Youden index.

c HD: hemodialysis.

d *P*<.05.

### Diagnostic Performance of Foot Temperature Parameters in Predicting the Presence of Severe PAD

ROC analysis was performed to further evaluate the diagnostic utility of various temperature parameters for identifying severe PAD (Table 5). The temperature difference between the wrist and the foot at the end of the HD session emerged as a significant indicator of arterial flow abnormalities. Specifically, for the detection of abnormal distal PTA flow, the wrist-foot temperature difference at the sole yielded an AUC of 62.0% (95% CI 55.2%‐68.5%; Figure 4). At the instep, this parameter showed an AUC of 59.1% (95% CI 52.3%‐65.7%). Notably, using a cutoff value of –2.2 °C or less for the wrist-foot difference at the instep provided a high specificity of 97.6% (95% CI 94.0%‐99.3%) for identifying abnormal PTA flow. The temperature parameters demonstrated even greater discriminative power for abnormal distal ATA flow. The wrist-foot temperature difference at the end of dialysis showed an AUC of 64.5% (95% CI 57.8%‐70.9%) at the sole and 62.0% (95% CI 55.2%‐68.5%) at the instep (Figure 5). Furthermore, absolute foot temperature at the end of the HD session was also a significant indicator of abnormal ATA flow, with an AUC of 64.5% (95% CI 57.7%‐70.8%) at the sole and 61.5% (95% CI 54.7%‐68.0%) at the instep. According to Youden index, a sole temperature cutoff of greater than 30.44 °C at the end of HD yielded a sensitivity of 70.8% (95% CI 55.9%‐83.0%) and a specificity of 58.2% (95% CI 50.4%‐65.7%) for detecting abnormal ATA flow (Table 5).

**Figure 4.**
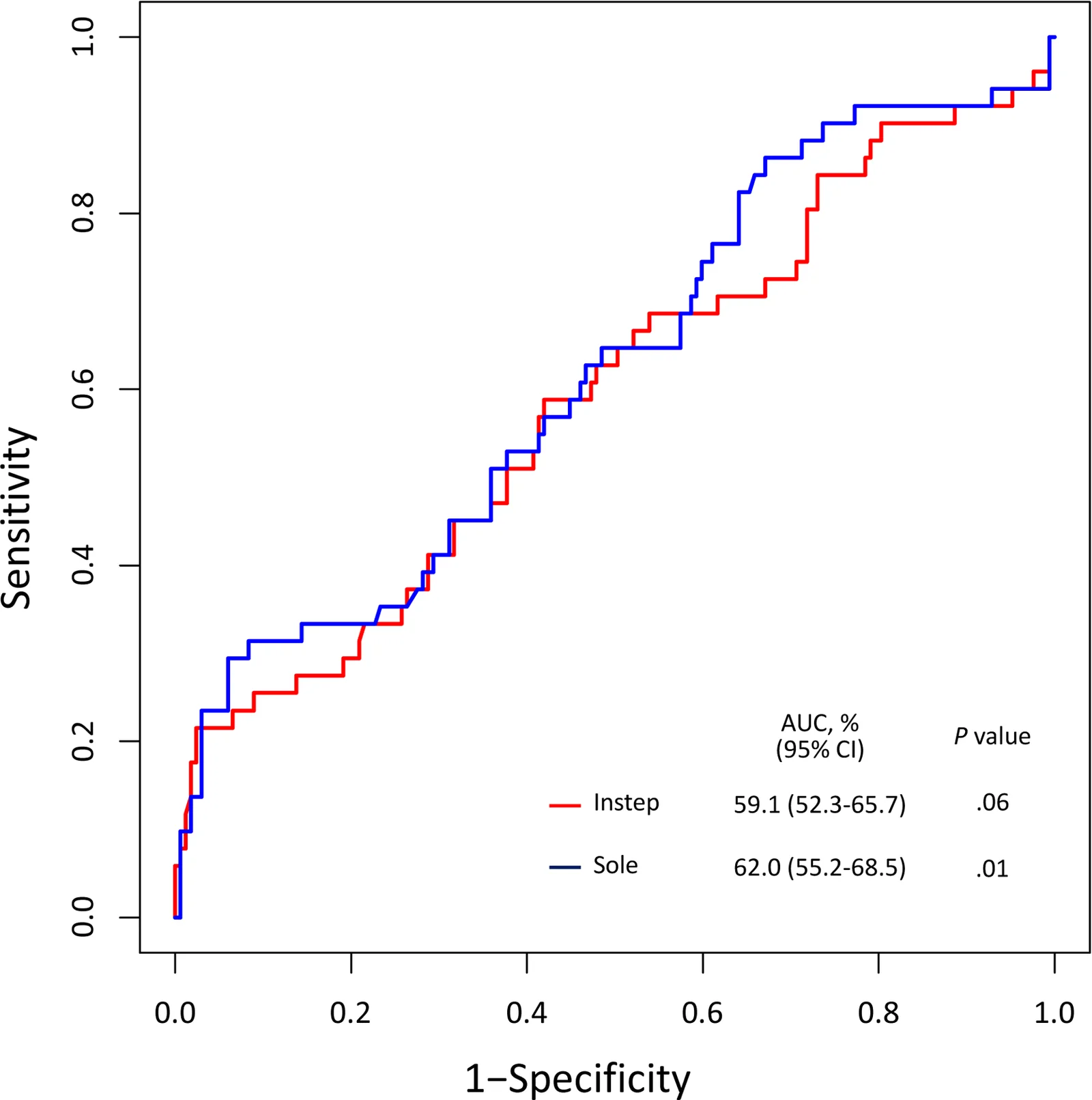
Receiver operating characteristic curves of wrist-to-foot temperature differences measured at the end of the hemodialysis session, at the instep and sole, for the discrimination of abnormal distal posterior tibial artery (PTA) flow. AUC: area under the curve.

**Figure 5.**
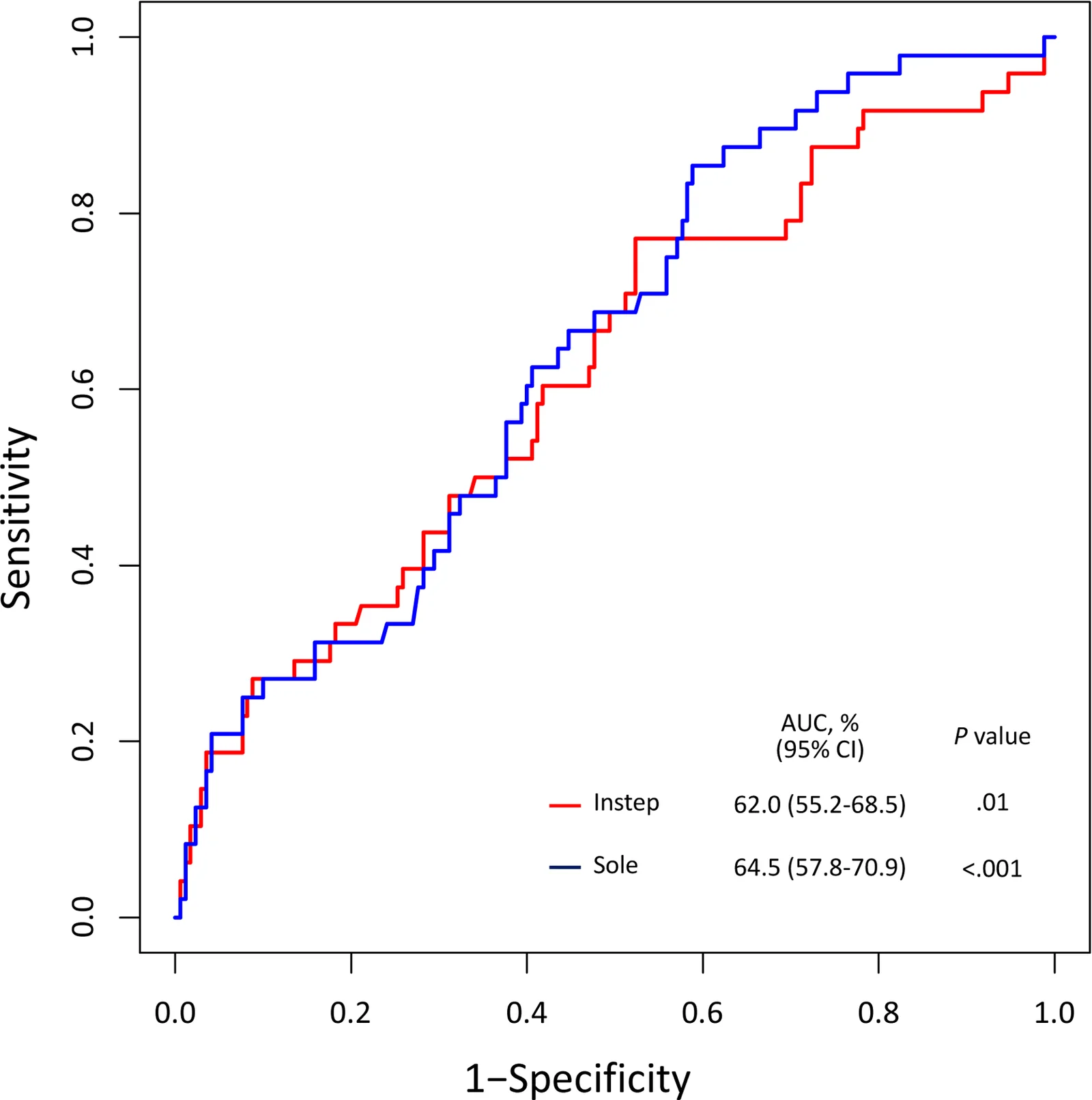
Receiver operating characteristic curves of the wrist-to-foot temperature differences measured at the end of the hemodialysis session, at the instep and sole, for the discrimination of abnormal distal anterior tibial artery (ATA) flow. AUC: area under the curve.

**Table 5.** Diagnostic value of temperature parameters for distinguishing between normal and abnormal distal posterior tibial artery (PTA)/anterior tibial artery (ATA) flow.

Outcome/position/parameter	AUC^a^, % (95% CI)	Cutoff (°C)^b^	Sensitivity, % (95% CI)	Specificity, % (95% CI)
Abnormal distal PTA flow				
Instep				
Temperature difference between the wrist and foot at the end of the HD^c^ session	59.1 (52.3‐65.7)^d^	≤–2.2	21.6 (11.3‐35.3)	97.6 (94.0‐99.3)
Temperature difference between the ear and foot at the end of the HD session	57.3 (50.5‐64.0)^d^	≤4.5	54.9 (40.3‐68.9)	67.1 (59.4‐74.1)
Foot temperature before the HD session	52.7 (45.9‐59.5)	—^e^	—	—
Foot temperature at the end of the HD session	57.2 (50.4‐63.9)^d^	>31.92	51.0 (36.6‐65.2)	71.9 (64.4‐78.5)
Sole				
Temperature difference between the wrist and foot at the end of the HD session	62.0 (55.2‐68.5)^d^	≤–1.2	29.4 (17.5‐43.8)	94.0 (89.3‐97.1)
Temperature difference between the ear and foot at the end of the HD session	59.4 (52.6‐66.0)^d^	≤4.87	52.9 (38.5‐67.1)	67.7 (60.0‐74.7)
Foot temperature before the HD session	52.8 (45.9‐59.6)	—	—	—
Foot temperature at the end of the HD session	59.4 (52.5‐65.9)^d^	>30.44	66.7 (52.1‐79.2)	57.5 (49.6‐65.1)
Abnormal distal ATA flow				
Instep				
Temperature difference between the wrist and foot at the end of the HD session	62.0 (55.2‐68.5)^d^	≤1.94	77.1 (62.7‐88.0)	47.7 (39.9‐55.4)
Temperature difference between the ear and foot at the end of the HD session	61.5 (54.7‐68.0)^d^	≤4.5	60.4 (45.3‐74.2)	68.2 (60.7‐75.2)
Foot temperature before the HD session	50.2 (43.4‐57.1)^d^	>29.35	81.3 (67.4‐91.1)	32.4 (25.4‐39.9)
Foot temperature at the end of the HD session	61.5 (54.7‐68.0)^d^	>31.16	66.7 (51.6‐79.6)	61.8 (54.0‐69.1)
Sole				
Temperature difference between the wrist and foot at the end of the HD session	64.5 (57.8‐70.9)^d^	≤2.88	85.4 (72.2‐93.9)	41.2 (33.7‐49.0)
Temperature difference between the ear and foot at the end of the HD session	63.8 (57.1‐70.2)^d^	≤4.87	58.3 (43.2‐72.4)	68.8 (61.3‐75.7)
Foot temperature before the HD session	53.8 (46.9‐60.6)	—	—	—
Foot temperature at the end of the HD session	64.5 (57.7‐70.8)^d^	>30.44	70.8 (55.9‐83.0)	58.2 (50.4‐65.7)

a AUC: area under the curve.

b According to the Youden index.

c HD: hemodialysis.

d *P*<.05.

e Not applicable

Lastly, we performed DeLong test to examine whether the addition of temperature monitoring yields incremental diagnostic value over the baseline ABI (Table 6). Regarding abnormal distal PTA flow, temperature parameters at both the instep and sole failed to offer incremental diagnostic value over an ABI value of less than 0.9. Conversely, relatively stronger diagnostic utility was observed for abnormal distal ATA flow. For the instep measurements, although the improvements did not achieve statistical significance, the ΔAUC values increased by roughly 1% (95% CI –5.5% to 7.5%) to 4% (95% CI –2.1% to 10.1%). Notably, for the sole measurements, the wrist-to-foot temperature difference at the end of dialysis yielded a significant increase in AUC (ΔAUC 5.6%, 95% CI 0.2%-11.0%). Additionally, a potential trend toward improved AUC was noted for foot temperature at the end of the HD session (ΔAUC 5.1%, 95% CI –1.0% to 11.2%).

**Table 6.** Added predictive value of foot temperature parameters over ankle-brachial index (ABI) value of less than 0.9 for abnormal distal posterior tibial artery (PTA) and anterior tibial artery (ATA) flow.

Outcome/position/parameter	ABI <0.9 alone (95% CI)	Temperature+ABI <0.9 (95% CI)	ΔAUC^a^, % (95% CI)	*P* value
Abnormal distal PTA flow				
Instep				
Temperature difference between the wrist and foot at the end of the HD^b^ session	69.4 (61.6-77.1)	70.4 (60.3-80.5)	1.0 (–5.5 to 7.5)	.76
Temperature difference between the ear and foot at the end of the HD session	69.4 (61.6-77.1)	70.1 (59.9-80.2)	0.7 (–5.7 to 7.0)	.84
Foot temperature before the HD session	69.4 (61.6-77.1)	71.3 (61.3-81.2)	1.9 (–4.9 to 8.6)	.58
Foot temperature at the end of the HD session	69.4 (61.6-77.1)	69.9 (59.6-80.2)	0.5 (–6.0 to 7.1)	.88
Sole				
Temperature difference between the wrist and foot at the end of the HD session	69.4 (61.6-77.1)	71.4 (61.6-81.3)	2.0 (–4.4 to 8.4)	.53
Temperature difference between the ear and foot at the end of the HD session	69.4 (61.6-77.1)	70.6 (60.8-80.4)	1.2 (–4.7 to 7.1)	.69
Foot temperature before the HD session	69.4 (61.6-77.1)	68.0 (57.6-78.5)	–1.3 (–8.0 to 5.3)	.69
Foot temperature at the end of the HD session	69.4 (61.6-77.1)	70.3 (60.3-80.4)	0.9 (–5.3 to 7.2)	.77
Abnormal distal ATA flow				
Instep				
Temperature difference between the wrist and foot at the end of the HD session	71.0 (62.9-79.2)	75.0 (65.7-84.4)	4.0 (–2.1 to 10.1)	.19
Temperature difference between the ear and foot at the end of the HD session	71.0 (62.9-79.2)	74.8 (64.8-84.8)	3.7 (–2.7 to 10.2)	.26
Foot temperature before the HD session	71.0 (62.9-79.2)	72.1 (62.1-82.2)	1.1 (–5.2 to 7.4)	.73
Foot temperature at the end of the HD session	71.0 (62.9-79.2)	74.5 (64.3-84.7)	3.5 (–3.2 to 10.1)	.31
Sole				
Temperature difference between the wrist and foot at the end of the HD session	71.0 (62.9-79.2)	76.6 (68.0-85.2)	5.6 (0.2 to 11.0)	.04
Temperature difference between the ear and foot at the end of the HD session	71.0 (62.9-79.2)	76.3 (67.0-85.5)	5.2 (–0.6 to 11.1)	.08
Foot temperature before the HD session	71.0 (62.9-79.2)	73.7 (63.9-83.5)	2.6 (–3.8 to 9.0)	.42
Foot temperature at the end of the HD session	71.0 (62.9-79.2)	76.1 (66.7-85.6)	5.1 (–1.0 to 11.2)	.10

a AUC: area under the curve.

b HD: hemodialysis.

## Discussion

### Principal Findings

In this prospective cohort study of patients with ESRD undergoing maintenance HD, we explored the diagnostic potential of intradialytic foot temperatures for detecting severe PAD. First, we found that in this population, where arterial calcification is prominent, the ABI may remain near-normal despite the presence of severe PAD as confirmed by DUS. Second, absolute foot temperatures at the end of the dialysis session were significantly higher in patients with severe ATA disease compared with those without. Finally, the temperature gradient between the wrist and foot served as a significant predictor of severe ATA or PTA disease, achieving an AUC of up to 64.5% (95% CI 57.8%-70.9%) for abnormal ATA flow. These results suggest that monitoring foot temperatures during HD provides an accessible and noninvasive means of screening for PAD in the vulnerable ESRD population. Nonetheless, the discriminative ability of these temperature parameters was modest (AUCs of approximately 0.59‐0.65), and because all estimates were derived and tested within the same cohort without internal or external validation, these findings should be interpreted as demonstrating an association rather than establishing a standalone diagnostic tool.

The diagnosis of PAD in patients with ESRD remains a significant clinical challenge due to the inherent limitations of conventional screening tools. The ABI, while widely used as a first-line diagnostic test, frequently yields falsely elevated or “normal” results in the HD population [17,18]. This is due to extensive arterial calcification, which renders the crural vessels noncompressible and prevents the accurate measurement of distal blood pressure [8]. This leads to a high rate of underdiagnosis in a population that is otherwise at extreme risk for CLTI and amputation. Furthermore, renal insufficiency is frequently associated with a more distal, crural distribution of atherosclerosis, which further complicates pressure-based assessments that are more sensitive to proximal disease. In our cohort, limbs confirmed to have severe PAD via DUS still exhibited mean ABI values as high as 0.89 (SD 0.27) to 0.90 (SD 0.26), suggesting that relying on ABI alone would have missed a substantial proportion of patients with severe tibial artery disease. We also demonstrated the feasibility of detecting severe PAD through the temperature gradient between the wrist and foot at the end of the HD session. For example, an instep wrist-foot temperature difference cutoff of –2.2 °C or less achieved a remarkable specificity of 97.6% (95% CI 94.0%-99.3%) for detecting abnormal flow in the PTA. By leveraging the hemodynamic stress during the dialysis process, this method provides a functional assessment of perfusion that is independent of vessel wall compressibility. Intradialytic thermography offers a potential screening adjunct, identifying high-risk patients who would otherwise be cleared by traditional, yet possibly less reliable, pressure-derived measurements.

Based on the established relationship between thermodynamics and hemodynamics, the use of skin temperature during HD as a proxy for arterial perfusion provides a novel physiological entry point for detecting PAD in patients with ESRD. Skin temperature has been previously recognized as an indicator of cutaneous blood flow, with infrared thermography and contact sensors being used to map ischemic zones in high-risk populations [19-21]. Prior studies have demonstrated that abnormal skin temperatures in the feet correlate significantly with reduced ABI values and abnormal limb arterial ultrasonography [20,22]. Our team previously reported that a significant postprocedural increase in foot temperature serves as a robust predictor of wound healing after EVT [16], reinforcing that thermal changes can directly reflect perfusion. However, interpretation of foot temperatures varies in different settings and can be challenging. In patients with DM, focal increases in foot temperatures are predictive of ulceration [15,23], while globally warmer feet suggest autosympathectomy related to diabetic neuropathy, with subsequent arteriovenous shunting and increased blood flow to the skin [19,22]. Thus, absolute foot temperature alone may be confounded and insufficient to reflect PAD severity. Similar to the use of myocardial stress testing to unmask underlying CAD, this present study advances this diagnostic concept by leveraging HD as a “thermal stress test” for the peripheral vasculature. To ensure diagnostic precision, the temperature at the wrist of the nonaccess arm served as a proximal, well-perfused reference. This approach allowed for the isolation of local thermal changes elicited specifically by vascular stress, thus controlling for systemic temperature fluctuations related to the dialysate itself. We found that while baseline temperatures before dialysis were similar between groups, the temperature at the end of the session significantly differentiated those with severe PAD. Specifically, the wrist-foot difference at the sole effectively discriminated abnormal flow in both the PTA and ATA. Interestingly, it is a lack of temperature decrease during HD that identifies severe PAD in our cohort (ie, those with severe PAD have higher foot temperatures). This paradoxical finding conforms with literature [18,19], suggesting disrupted vasoconstriction mechanisms in these patients. Our study demonstrates that wearable mHealth sensors provide a physiological foundation for future research into the underlying mechanisms of PAD in patients with ESRD.

Socioeconomic disparities significantly delay the diagnosis of PAD, leading to higher amputation rates in underserved populations [24]. Patients with ESRD, in particular, are frequently overlooked, with limited access to specialized care due to ambulatory difficulties and socioeconomic disadvantages [7]. Traditional screening for PAD via ABI or DUS is often limited by accessibility, resulting in a reactive approach where referral is initiated only after ulcers or gangrene appear. Intradialytic foot temperature monitoring offers a solution to overcome these barriers by using the Temp Pal, a wearable mHealth thermometer system that provides high-fidelity, continuous monitoring with minimal overhead. While studies have demonstrated the feasibility of smart-socks [21,25], such specialized apparel is unavailable in many regions, including Taiwan. In contrast, cutaneous thermometers are significantly cheaper, highly accessible, and reusable, making them a more practical choice for global implementation. More importantly, integrating this monitoring method into routine dialysis sessions allows patients to undergo screening without additional travel or time commitments, ensuring accessibility to those with limited mobility or resources. Our ultimate goal is to mitigate health disparities within the high-risk population with ESRD, thereby improving both the trajectory of vascular disease and long-term survival. These potential benefits, improved screening access, reduced disparities, and earlier identification, were not evaluated in the present study and remain hypotheses to be tested in future implementation and outcome studies.

### Limitations

While this study offers a novel approach to PAD screening, several limitations must be acknowledged. First, the sample size of 109 patients is relatively small and was recruited from 2 tertiary centers within a single geographic region. As our cohort consisted exclusively of an East Asian population in Taiwan, the generalizability of our findings to other ethnic groups remains uncertain. Given that racial and socioeconomic disparities significantly influence the presentation and progression of CLTI, further research in more diverse populations is necessary to validate our findings. Second, we used DUS flow patterns at 5 cm above the ankle as our gold standard for defining severe PAD. Although DUS is a clinically standard and reliable tool for assessing arterial patency, it primarily evaluates macrovascular flow. We did not incorporate other perfusion markers, such as transcutaneous oxygen tension or skin perfusion pressure, which might have provided a better understanding of how intradialytic temperature shifts correlate with actual tissue oxygenation. Finally, the sensitivity of intradialytic temperature monitoring for detecting severe tibial artery flow abnormalities varied considerably across parameters and measurement sites, with a reciprocal trade-off in specificity. In addition, this study was not designed to assess long-term outcomes. Although early detection is critical in PAD, there is a lack of evidence that this screening method leads to improved limb salvage rates or survival benefits. Future longitudinal studies are required to determine if initiating early interventions based on these thermal warning signs can successfully reduce major adverse limb events, major adverse cardiovascular events, or mortality associated with PAD in the population with ESRD. In addition, as most patients were asymptomatic and were screened irrespective of symptoms, formal clinical PAD staging (eg, Fontaine or Rutherford categories) was not performed; disease severity was instead defined objectively by DUS flow patterns. Moreover, several factors may have affected the accuracy of our temperature measurements: the ambient temperature of the dialysis unit was not systematically recorded, the access (fistula) arm was not measured, and a single nonaccess arm was used as the wrist reference for both legs, which could introduce bias if that reference limb itself had impaired perfusion.

### Conclusions

This study demonstrated that intradialytic foot temperature monitoring is a potential diagnostic adjunct for detecting severe PAD in patients with ESRD. We found that in patients with ESRD, the ABI can remain near-normal despite severe disease, whereas the wrist-foot temperature difference at the end of the HD session was significantly associated with abnormal tibial flow, although sensitivity and specificity varied considerably across temperature parameters. Temperature monitoring with mHealth devices during HD may provide an accessible, low-cost screening solution that mitigates health disparities in patients with ESRD. Further longitudinal studies are required to confirm the survival benefits of this novel method. Because the observed discriminative ability was modest and has not yet been externally validated, intradialytic temperature monitoring should be regarded as a complementary adjunct to, rather than a replacement for, established vascular assessments such as the ABI.

## Abbreviations

ABI: ankle-brachial index
ATA: anterior tibial artery
AUC: area under the curve
CAD: coronary artery disease
CLTI: chronic limb-threatening ischemia
DM: diabetes mellitus
DUS: duplex ultrasound
ESRD: end-stage renal disease
EVT: endovascular therapy
GEE: generalized estimating equation
HD: hemodialysis
IRB: Institutional Review Board
mHealth: mobile health
OR: odds ratio
PAD: peripheral artery disease
PTA: posterior tibial artery
ROC: receiver operating characteristic
